# Iodide of Potassium in Diseases of the Brain in Children

**Published:** 1860-09

**Authors:** John Coldstream


### IODIDE OF POTASSIUM IN DISEASES OF THE BRAIN IN CHILDREN.

BY DR. JOHN COLDSTREAM.

Twenty years ago iodide of potassium was recommended by Roeser, as a remedy of special power in hydrocephalus, and since this time Drs. Risdon, Bennett, Copland, Evanson, Maunsell, Willshire, West, Wood, of Philadelphia and others, have added their testimony as to the value of this medicine in this and analogous cases.

"My own experience," writes Dr. Coldstream, "has gradually led me, for a considerable time past, to its employment, almost exclusively, in the treatment of those numerous ailments of children, which we cannot but regard as indicative of a tendency to hydrocephalus. In all cases in which, from the course of symptoms, I have reason to believe that the central organs of the nervous system, or their envelopes, are affected with strumous inflammation, (tubercular cerebritis, or meningitis,) or its consequences, after moderate purging, and perhaps application of leeches to the head, I am in the habit of prescribing the iodide, in doses of from half a grain to three grains, every three or four hours, generally dissolved in some carminative water, and continuing it in doses, varied according to the symptoms, for many days, or even until convalescence is fully established; and I am quite satisfied that, under this treatment, with the occasional addition of blisters to the shaven scalp, I have seen far more prompt and decided effect produced upon the disease than I used to see under the old treatment.

"When opportunities have been afforded of commencing the use of the iodide early, it has appeared in several cases to arrest the progress of the disease rapidly, so that the formidable effects of effusion, indicated by squinting and convulsions, have not supervened. In less favorable circumstances, in cases where considerable prostration had succeeded to great febrile action, and in which starting and squinting had become prominent symptoms, I have seen, in not a few instances, the free use of iodide of potassium followed by amendment and complete recovery. In such cases, and in others still further advanced, I have given larger doses, even to the extent of four grains, several times a day, to children of from four to eight years of age.

"The medicine is very seldom refused by the patient, and I cannot say that I have ever seen it either increase the nausea that so frequently exists in the earlier stages of thé disease, or produce any other untoward effect; especially have I have never seen it induce salivation, which the drug sometimes seems to cause when given for other ailments.

"It seems generally to act upon the kidneys; yet I cannot say that the amount of relief to the head symptoms bears any very obvious relation to the quantity of urine excreted.

"Although I have no doubt that the iodide is more especially useful in cases where there exists more or less of the scrofulous diathesis, I have often used it with satisfaction in patients apparently free from all such taint; even in cases where the ailment seemed to have followed injury from external violence, as so often happens in young children. I am not prepared, however, to assert that the iodide is more useful than calomel in *all* cases of inflammation of the brain and its appendages. When we have to treat robust and full-blooded children, in whom there is good reason to believe that the threatened disease of the nervous system stands more or less directly connected with preceding disorder of the digestive organs, I have no doubt of the superior efficacy of the mercurial treatment, combined with antimonials and salines; but when, after having duly administered these, symptoms of cerebral disorder continue, I would have recourse to the use of the iodide.

"In cases of convulsions from teething, which, amongst ill-fed children, living in badly aired localities, are not unfrequently followed by hydrocephalus, I have used the medicine with much satisfaction.

"I have occasionally employed the proto-ioduret of mercury, as advised by Evanson and Maunsell, but not with more obvious benefit than I have been accustomed to see resulting from the use of the iodide of potassium. During convalescence, I generally prescribe the iodide of iron; sometimes a vegetable tonic, combined with the iodide of potassium.

"In several cases of recovery from severe attacks of meningitis, it has occurred to me to find the mental powers of the little patients considerably impaired. This result has occasionally been protracted for many years, and seems likely to prove permanent; but generally it has become less apparent, and ultimately passed off entirely.

"In thus endeavoring to recall attention to what I believe to be a truly valuable agent in the treatment of a class of formidable diseases, I would not overlook the fact, that all past experience tends to assure us that a great majority of cases of disease of the brain in early life, prove fatal under all kinds of treatment. In advanced stages of the tubercular forms of these diseases, we may not yet venture to hope for any great advantage in the use of the iodide of potassium. But I am disposed to agree with Drs. Copland, Willshire, and West, in believing that they may be cut short, if subjected to treatment in an early stage, more frequently than is generally imagined. My own experience leads me to regard the iodide as more likely than any other drug to promote the desired end; and my confidence in it, as *the* remedy best adapted to all stages of the tubercular diseases of the head, is so strong, that whatever else might be done, or left undone, I would persevere in administering it, even in circumstances the most desperate."—*Edinburgh Medical Journal, Dec.* 1859.

---

☞ Lord Bacon remarked: "Certainly every medicine is an innovation, and he that will not apply new remedies must expect new evils, for time is the greatest innovation; and if time, of course, alter all things for the worse, and wisdom and counsel shall not alter them for the better, where shall be the end?"

